# Being done with “it”: forensic psychiatric patients’ experiences with the development and treatment for co-occurring substance use disorders and mental disorders

**DOI:** 10.1186/s12888-025-07762-8

**Published:** 2026-01-09

**Authors:** Johan Green, A. S. Lindqvist Bagge, E. Punzi, P. Andiné, M. Wallinius, M. Hildebrand Karlén

**Affiliations:** 1 Centre for Ethics, Law and Mental Health, Department of Psychiatry and Neurochemistry, Institute of Neuroscience and Physiology, Sahlgrenska Academy, University of Gothenburg, Gothenburg, Sweden; 2 Department of Psychology, University of Gothenburg, Gothenburg, Sweden; 3 Department of Social Work, University of Gothenburg, Gothenburg, Sweden; 4 Department of Forensic Psychiatry, National Board of Forensic Medicine, Gothenburg, Sweden; 5 Forensic Psychiatric Clinic, Sahlgrenska University Hospital, Gothenburg, Sweden; 6 Psychiatry, Department of Clinical Sciences Lund, Lund University, Lund, Sweden; 7 Research Department, Regional Forensic Psychiatric Clinic, Växjö, Sweden

**Keywords:** Forensic psychiatry, Co-occurring disorders, Substance use disorder, Qualitative research, Interpretative phenomenological analysis, Patient-centred care, Identity transition

## Abstract

**Background:**

Co-occurring substance use disorders, and mental disorders are associated with significant suffering, high risks of violent reoffending and relapse in both substance use and psychotic episodes. Treatment for co-occurring substance use disorders in forensic mental health settings would benefit from incorporating patients’ understanding of their disorders.

**Method:**

Using an interpretative phenomenological analysis, this study explored and interpreted the experiences of 13 Swedish forensic psychiatric patients regarding the development of, and being treated for, co-occurring disorders.

**Results:**

The analysis yielded four experiential themes: “substituting a missing tool”, “’it’: a self-sustaining ecosystem”, “treated but not cared for”, “comprehending fragments of the self”. Results illustrated how substance use, mental and physical health and social worlds fused into an interconnected ecosystem (referred to as “it”) where negative developments in any domain risked inducing relapse. Diagnosis-focused treatment was perceived as disconnected from this reality, leading to selective censorship and treatment disengagement. In understanding their past, patients experienced a fragmentation between their identities resulting in the conceptualisation of an “addict self” who inhabited “it” and who was separate from their current, “moral self”.

**Conclusion:**

Flexible treatment approaches that address early-life trauma and that assist in integrating patients’ past actions, present vulnerabilities, and future potential are beneficial to the forensic mental health services.

**Clinical trial number:**

Not applicable.

## Background

Co-occurring mental disorders (MD) and substance use disorders (SUD) (henceforth referred to as COD) pose significant challenges to treatment in the forensic mental health services (FMHS). Patients with COD in FMHS typically have poor treatment outcomes [1] and increased risks of relapse and criminal recidivism [2]. Treatment of FMHS patients is further complicated by additional challenges, including compounded adverse childhood experiences [3, 4], high rates of self- and other-directed violence and suicidality [5], neurocognitive dysfunction [6], and patients’ limited insight into their conditions [7].

Given these challenges, there is a pressing need for increased evidence-based practices for treating COD [8, 9]. As a part of this call for action, the benefits of aligning treatment practices with patients’ comprehension and experiences of, their conditions have been highlighted [10, 11]. Lack of insight of a disorder [12] could have neurobiological underpinnings in prefrontal lobe dysfunction and is particularly pronounced in patients with schizophrenia [13]. Simultaneously, impaired insight has been linked to increased disorder severity, lower treatment compliance, more frequent rehospitalizations and higher risk of violent behaviour among people with a severe MD [14]. Given the severe pathology and heterogeneity characterising the COD forensic psychiatric patient group [15, 16], a deeper understanding of COD patients’ understanding of their disorders is warranted.

In the context of contemporary addiction treatment, conceptual models of addiction are often reduced to a dichotomy of volition/compulsion where the “addicted state” is characterised as a disorder of compulsion [17]. Individuals suffering from SUD are therefore often faced with the near irreconcilable dilemma of surrendering to two diametrically opposite yet equally true propositions: being addicted to a substance involves a loss of control over one’s actions, and the only way to reclaim control is through intentional effort [18]. This simplistic framework, however, inadequately captures the complex aetiology of SUD and COD. Modern theories highlight the interconnectedness of biological, psychological and social factors [19, 20] and treatment approaches for SUD are frequently recommended to incorporate early life trauma, social marginalisation, and socioeconomic constraints for several, non-forensic service groups [21]. Treatment that refrain from recognizing this pluralistic aetiology and instead reduce SUD to the determinism-voluntarism dichotomy risk coercing patients’ self-comprehension into one of two extremes: either viewing themselves as biomechanically compelled [22] and defining successful treatment outcomes in terms of external control, or as viewing themselves as volitional and capable of change but also as solely responsible for morally condemnable behaviour [23]. Both of these polarised perceptions of agency are unconducive to compulsory treatment in the FMHS. Increased insights into how patients with COD navigate this dilemma and whether their understanding aligns with or transcends the determinism-voluntarism dichotomy would help in developing person-centred, trauma-informed treatment approaches.

In Sweden, offenders with a severe MD are sentenced to treatment in FMHS rather than prison. Most (87% of male, 75% of female patients) have special court supervision, meaning discharge depends on demonstrating reduced risk of reoffending rather than completing a fixed treatment period [24]. The average treatment period is 7.5 years but longer for patients with SUD [24, 25]. Throughout treatment, patients are ideally offered a chance to explore and redefine their understanding of their disorder(s) and their inner processes. Prior qualitative interview studies impart that substance users’ shedding of their former selves and the donning of a new identity is a necessary – albeit insufficient – component to recovery [26–29]. The neogenesis of the self in compulsory treatment is invariably shaped in collaboration with the treatment staff [30]. Andersen [31] demonstrated that within SUD treatment institutions, patients’ narratives of their disorder must conform to local interpretations of change and that treatment staff frequently edit patients’ conceptions of their disorder. Given that treatment staff in FMHS may hold divergent stances in the SUD etiological discourse and sometimes adopt a punitive stance towards the disorder [32], the co-creation of meaningful patient narratives risks being negatively altered, superficially explored, or result in internalised self-stigmatisation, all of which are unconducive to treatment engagement [33].

Within mental health services in general, institutional authority over the conceptualisation of psychopathology and formulation of treatment needs risks disincentivising or barring treatment-seeking individuals whose own insight into their mental health and associated treatment needs differs from those imposed by the institution (see “Amanda” [31]). In Swedish FMHS, institutional authority might be more pervasive since discharge is often not contingent on fixed treatment periods, but on a review board’s assessment of the patient’s treatment progression and current risk for relapse and criminal recidivism. This model, while designed to enable adequate treatment and to protect society from potentially violent individuals, may incentivise patients to present a façade that would improve the board’s assessment of them [34]. Furthermore, the coercive nature of FMHS offers little leeway for patient autonomy and involvement in their mental health progression [35, 36]. Having patients censor themselves or having their understanding of their treatment needs edited by an institutional powerhouse risks making psychological mechanisms driving substance use relapse inaccessible for treatment [37].

Understanding how COD patients in FMHS make sense of their disorders and their experiences with treatment – past, present, and future – would provide valuable insights for a field in need of treatment direction in the form of increased patient involvement [11]. Therefore, the present study aimed to explore and interpret FMHS patients’ experiences with their COD, specifically focusing on their (past) experiences with the two disorders’ separate and joint development, their (present) experiences with being a patient with COD in forensic psychiatric treatment, and their (future) aspirations with respects to long-term treatment goals.

## Methods

This study was part of the FOR-SATA project on SUD assessment and treatment for FMHS patients in Sweden. The project was part of the FORevidence national initiative to enhance the evidence base practices in Swedish forensic psychiatry.

### Setting, participants and the population

This study is based on semi-structured interviews with patients from a large, high-security FMHS clinic in Sweden which in 2019 implemented new SUD focused assessment and treatment guidelines [32]. Two research assistants employed at the clinic identified potential participants based on eligibility criteria: having a history of substance use, having undergone an SUD assessment as part of the new guidelines, and Swedish language proficiency. Before each recruitment attempt, the patient’s treating physician was first consulted to assess the patient’s capacity to provide informed consent. Patients were deemed ineligible if they exhibited acute psychotic symptoms, insufficient cognitive functioning, or posed a significant risk for violence. For those considered eligible for recruiting, a semi-purposive sampling method was used to approximate the national population of COD patients in the FMHS in terms of primary MDs, substance use, gender, and severity of psychopathology [24]. The recruited patients’ current mental status and emotional stability were considered when recruiting and planning interviews. All participants provided verbal and written consent prior to being interviewed and received a small compensation (approx. € 9) for their time. For security reasons, staff remained immediately outside the interview room, and the interviewer carried a personal alarm.

A total of 13 patients (10 men and three women) were interviewed. All interviews were recorded and transcribed and varied between 22 and 81 min in length (mean = 46 min). Excerpts from the transcripts have been translated from Swedish to English by the authors. All identifiable information has been excluded from the transcripts.

While the participants’ exact history of substance use, MDs and other descriptive data cannot be presented due to the risk of identifying participants, the sample approximated the national population of Swedish FMHS patients with COD. National registry data reports that Swedish FMHS patients (*N* = 2070) are 85% male, have a median age of 39, that the most common primary diagnosis is schizophrenia followed by neurodevelopmental disorders, that 68% have a documented history of substance use where drug use was the most common, followed by alcohol and pharmaceuticals, and that 15% were considered to have insight into their disorder [24].

### Interview

Semi-structured interviews were conducted to focus on the participants’ experiences of their COD, the disorders’ development, and their treatment. The interview guide covered four main sections: (1) development of SUD, (2) development of COD, (3) past relationship with substance use, and 4) relationship with substance use during and after the treatment period. Each section began with an initial prompting question (e.g., “have you had any kind of mental or physical problems that you believe are connected to your substance use in any way?”) with several suggestions on follow-up questions (e.g., “what kinds of problems”, “to what extent and in what way have they negatively impacted you?“). The interviewer prompted the participants to elaborate when they provided brief answers (e.g., “tell me more”, “what exactly does that meant to you?”). The interviewer also shifted focus to whatever topics the participants elected to discuss. At the end of each section, summaries were provided allowing participants to add, edit, or withdraw input.

### Analysis

The interview data were analysed using interpretative phenomenological analysis (IPA) – an analysis method allowing researchers to explore and interpret how participants make sense of life events [37–40]. It is particularly suited for studying individuals’ interpretations of phenomena within complex social contexts such as the FMHS. Given the complexity and heterogeneity of the COD patient group, IPA’s ability to study both unique and common experiences was especially valuable.

The IPA is the product of two schools of thought: phenomenology and hermeneutics. Phenomenology, broadly defined, is the view that the inner, experiential world is unique to each individual and that how one experiences a phenomenon, more so than the objective properties of the phenomenon, governs behaviour [37]. The goal of phenomenological analysis is to study the phenomenon as it is experienced within the individual’s “life world” [41]. The IPA also acknowledges that the participants’ interpretation of their experiences with a phenomenon is further interpreted by the researcher (double hermeneutics [42]), and that the interview details shape the overall meaning of the participants’ experience, while the complete report contextualises those details (hermeneutic cycle, ibid.).

The analysis followed the guidelines outlined by Smith et al. [39]. First, the main analyst (JG) listened to and read the interviews to become familiar with the material, taking notes on preliminary ideas for later reference. The second read-through was an in-depth idiographic analysis. Exploratory notes were recorded in the margins concerning participants’ experiences (the whole) and diction (the details). Then, by reviewing the exploratory notes and the original transcript, experiential statements were formulated for each transcript that strove to capture the researcher’s interpretation of the participant’s experiences [39]. For each participant, these experiential statements were organised into a unique set of personal themes.

Next, moving from idiographic to nomothetic understanding of the participants’ experiences, commonalities in the unique personal themes were identified. These commonalities were organised into group level themes, meant to capture shared experiences between participants. Both personal and group themes were jointly developed by main analyst JG and co-authors MHK, ALB and EP. Extracts from the transcripts were identified to illustrate the meaning of each group theme. The results presented below are the final themes jointly developed by co-authors JG, EP, MHK, and ALB.

### Reflective statement

IPA demands rigorous reflexivity from the researcher [39]. Participants’ experiences with a phenomenon are situated in their unique inner world and the researcher’s attempts to interpret them are further filtered through their own, equally unique inner world. Neutralising preconceived notions (tabula rasa) is not possible and merely acknowledging them (mea culpa) is insufficient. Instead, validity in IPA can only be achieved by reigning in, reporting, and capitalising on one’s past experiences [43]. To this end, the main analyst (JG) kept a reflexivity log during the analysis. With a background in criminology and limited experience with forensic psychiatry, JG’s early interviews were skewed towards establishing chronology and structure. With experience, however, interviews aligned more with the participants’ comprehension of their COD. This evolution in approach was influenced by JG’s position as an outsider at the clinic, which had a dual impact. While clinical naivete made some dimensions in patients’ experiences inaccessible, it also may have facilitated open and uncensured disclosure regarding sensitive topics from participants. It is also possible that, lacking clinical expertise, JG’s questions appeared more genuine, further promoting an open interview atmosphere. Additionally, post-interview debrief sessions were held with supervisors to minimise the potential bias from the interviewer’s emotional reactions to difficult interview contents (e.g., past trauma or violent crimes) in both the interviewing and the analysis. Lastly, the collaborative analysis by co-authors JG, MHK, ALB, and EP facilitated the utilisation of the research team’s diverse backgrounds and experiences while also making evident and mitigating the influence of our individual biases.

## Results

**Fig. 1:**
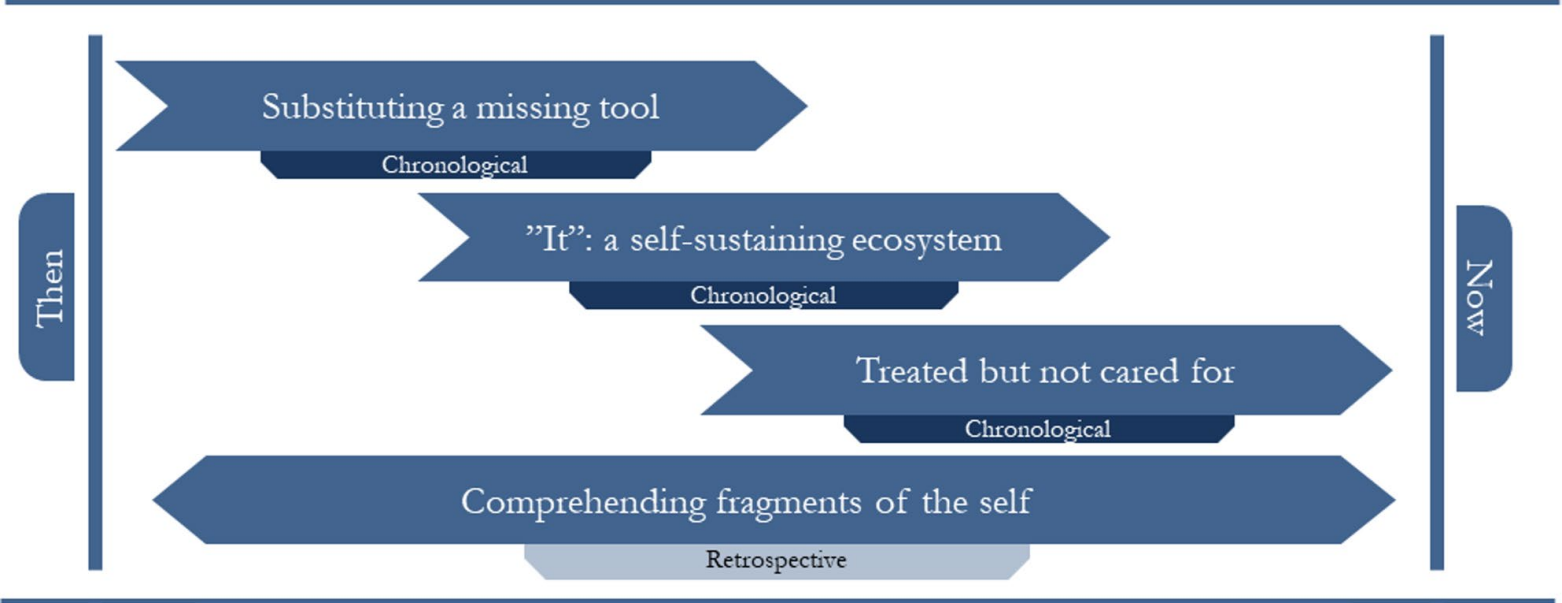
Conceptual structure of experiential themes

The analysis of the interview data yielded four themes: (1) *substituting a missing tool* which illustrates the perceived legitimacy of substance use in the participants’ lives; (2) *“It”: a self-sustaining ecosystem*, which describes how substance use was interconnected with other aspects of the participants’ lives; (3) *treated but not cared for*, outlining participants’ experiences with treatment; and (4) *comprehending fragments of the self* which demonstrates the participants’ struggles with making sense of their past and their future. The chronological progression of the participants’ experiences with the development of and treatment for COD (themes 1–3) and the overarching interpretative framework participants used to make sense of their experiences (theme 4) are visualised in Fig. 1. The prevalence of themes is presented in Table 1.

**Table 1:** Theme prevalence across participant transcripts

Participant	Theme			
	Substituting a missing tool	“It”: a self-sustaining ecosystem	Treated but not cared for	Comprehending fragments of the self
Ahmed	✔	✔	✔	✔
Benjamin	✔	✔	✔	✔
Charlie	✔	✔	✔	✔
David	✔	✔		✔
Ezekiel	✔		✔	✔
Finnegan	✔	✔	✔	✔
Gael	✔	✔	✔	✔
Harper	✔	✔	✔	✔
Ignacio	✔	✔	✔	✔
Jean	✔	✔		✔
Kimberly	✔	✔	✔	✔
Lenny		✔	✔	✔
Marlon	✔	✔	✔	✔

### Substituting a missing tool

This theme illustrates how the participants understood their substance use as serving a legitimate function, and the deficient mechanisms that legitimised it. At the surface of the participants’ reports lay a considerable variation in the reasons for using substances. Common in their experiences, however, was the perception that substance use served as a legitimate and irreplaceable tool in their lives.

Participants seemed to have constructed logical and defensible reasons for their substance use. Marlon, however, instantiated the belief that these reasons were justifications for satisfying cravings:

*No there’s no need for anything to happen. It’s not necessary. […] Blaming it on my cousin dying or my mom being sick or that my daughter is pissed at me*,* that’s not a reason. You take drugs because you like drugs (Marlon:41).*

Marlon, being an experienced drug user, gave voice to users far along in their addiction. He had first turned to drug use to regulate his own violent behaviour. Now, after his SUD had progressed extensively, the primary reason behind his drug use was simply manifold manifestations of cravings.

For others who had experienced significant trauma or who had had an early onset of an MD, substance use was perceived as a replacement for a cognitive or emotional tool they had not yet developed. The alleviation of dysphoria that substance use provided was a panacea in lieu of healthier, long-term options.

Finnegan’s opioid addiction stemmed from his attempts at pain management and had, throughout the years, repeatedly pushed him to the brink of suicide. He believed that his anxiety could only be managed artificially: “I used to think that [drug use] was the only solution there was” (Finnegan:08). In moments overcome by pain or anxiety, the only tool he conceived of to alleviate his distress was drug use.

Jean’s trauma and early-onset mood disorder was impossible for her to cope with at her young age. Consequently, she turned to self-harm and substance use for relief:

*As a distraction*,* yeah. But then it doesn’t go away simply because you hurt yourself. It is inside of me. But you think it it’ll ease the pain. That you’ll go somewhere else*,* away from yourself. But it’s still there. You have to medicate sometimes. That’s what I did (Jean:24).*

Jean’s story of being ill-equipped to deal with a significant mental burden and “going somewhere else” with drug use was a common one in this sample. Kimberly, sharing Jean’s experience of turning to drug use to cope with trauma, expanded on the missing psychological tools which the substance use replaced.

*Then I felt better – also*,* I could cope better with the fact that I felt bad. […] But I had an easier time talking about my issues when I was on amphetamines. So it also helped me open up and stuff. But today I can open up without drugs (Kimberly:10–14).*

Drug use did not only de-intensify the negative affect that Kimberly experienced, but she also felt that it allowed her to better communicate and manage her issues. In her transcript, Kimberly frequently emphasised how counselling had provided her with tools to manage her disorder constructively – tools that cannot be expected to be so developed in adolescence as to handle significant trauma or the burden of a serious MD.

David, having struggled to provide a consistent rationale for his continued drug use, arrived at a conjecture for an underlying reason:

*It wasn’t only that I*,* like*,* suppressed myself*,* it was more like*,* after the accident*,* I lost… it was also like a protest over how I had lost everything (David:05).*

David made sense of his drug use as a way to, in lieu of other means, signal to the world that he was falling apart. To him, substance use provided a tool to express or manage something that he struggled to comprehend.

Many whose substance use was tied to management of an MD highlighted the legitimacy of substance use vis-à-vis therapy, in that the urgency of their problems demanded “a quick fix” (Harper:18). Ahmed, who still considered drugs “[his] salvation” (Ahmed:07), believed that they served no other purpose but the legitimate management of his mental health.

*There were many options – other drugs. I was pretty anti-drugs once*,* so I saw [drug-use] as nothing but medication (Ahmed:09).*

While participants perceived substance use as a legitimate solution to an acute problem, they also seemed to have been aware of the price they would eventually have to pay. Gael proffered a reflection on the long-term consequences of opting for the short-term solution:

*I believe [substance use] has made [the disorder] worse. At the same time*,* I’ve also had a thought that I was actually trying to fix it and regulate it. But I believe that*,* in the end*,* it has made it worse. […] I mean the diagnosis has gotten worse. I feel that schizoaffective is a worse diagnosis (Gael:08).*

Trauma, or a burgeoning MD, proved to be an overwhelming burden during their formative years. The discovery that substance use could alleviate a dysphoria they struggled to even comprehend or name, provided a much-needed reprieve. Years later, many still defended the legitimacy of their choice, even as they recognised the problems it had caused.

### “It”: a self-sustaining ecosystem

This theme portrays participants’ experiences of how their substance use demanded more and more upkeep and how, eventually, their mental health, physical health, relationships, criminality, and all other facets of their lives became inexorably intertwined with, infected by, and in service of their substance use. The actions that were then required of them to sustain this ecosystem would ostracise them from their old world, embedding them deeper in “it”.

Many participants, once their addiction had blossomed, referred to an “it”, defined as a fully self-sustaining ecosystem comprised of interrelated domains: mental and physical health, substance use, friends, family, criminality, and more. Ignacio’s transcript illustrated well how drug-use had subsumed all aspects of his life and bound him to take actions to sustain it.

*I was constantly selling cannabis to finance my addiction. I didn’t have time for anything else. That was all I did*,* I sold*,* and I smoked (Ignacio:12).*

Marlon also frequently referred to an “it” and defined it as an intricate and deeply rooted system of mental health management, drug use and criminality.

*The psychosis is about: selling*,* arming yourself*,* making money*,* doing drugs*,* staying awake*,* not getting killed*,* yadda yadda*,* like I’m saying. A day in the life of a criminal (Marlon:36).*

In his explanation of “it”, Marlon transitioned from his experiences with psychosis, to his substance use, and to his criminal lifestyle suggesting their interconnectedness.

As the interdependent relationship between substance use and the MD developed, participants effectively became entrenched deeper in “it”. Their physical, psychological, and social worlds all became suffused with their substance use and would eventually demand criminal and sometimes violent actions of them to sustain it – so that it, in turn, could sustain them. Ignacio’s transcript provided an example of how the expansion of “it” came to demand violent criminal acts of him to sustain it:

*To me*,* human life has no value*,* so I don’t regret [the murder]. I don’t care. But I know it was all because of the drugs. If I hadn’t started doing drugs*,* my life would’ve been very different (Ignacio:18).*

Ignacio’s entrenchment in “it” required him to commit violent crimes to sustain it while the “unbelievable amounts” (Ignacio:09) of drugs he consumed was what gave him the capacity for the violence needed to sustain it. Most participants echoed Ignacio’s experience of this cyclical dependency: their substance use demanded criminal acts to sustain it, while their ability to function and commit said crimes depended on continued substance use.

Substance-induced violence eventually contributed to participants’ ostracisation and the loss of meaningful relationships. Friends and family were driven away, and new friends were recruited from within the substance-suffused ecosystem - spreading its influence. Lenny and Harper both provided examples of this process. Lenny used alcohol to cope with feelings of isolation, but he later described that drinking made him violent, and that his violent behaviour eventually drove people away, worsening his isolation and embedding him deeper in “it”. Harper, whose transcript was heavily influenced by finding a community to replace her broken family, described being left behind by her old friends and remaining with her addicted peers:

*I only hung out with my addict group. Regular friends disappeared. They moved on in life*,* quit pot and such*,* got an education. I didn’t (Harper:29).*

She described how members of this community supported each other, but turned adversarial as soon as someone suggested they wanted to quit: “They got jealous if you ever thought of quitting, they did not want that” (Harper:30). For Harper, her much-sought-after feeling of belonging became contingent on her sustained drug use. Her social life became another part of “it”, threatening social rejection should she quit drugs.

When their substance use habit had grown to a point where the ecosystem strained to support it, some became aware of a shift in what the substance use provided: it was no longer the cure for their ailment but the cause of it. David described how the function the drugs served inverted:

*So that… you’ve tried to enjoy it*,* but you’ve only felt worse. So*,* you’ve done even more to feel good*,* but you’ve only felt even worse (David:10).*

He compared this suddenly negative reaction to drug use with an allergic reaction: “it’s like I can’t tolerate it anymore” (David:08). David described his attempts to quit but that, since his substance use had tethers in his biological, psychological and social worlds, quitting was a matter of letting your entire life – “it” – fall apart. Being sentenced to FMHS became the pinnacle of the fall.

The participants experienced, in one way or another, that the desire to quit had only a minor impact on their ability to quit. Harper expressed that, after her first overdose, she saw where her habit was leading her but knew that she was incapable of quitting.

*Yeah*,* after the first overdose I got scared. So I thought*,* no now I’m really quitting. But then I barely made it out of the hospital before my next hit (Harper:26).*

She questioned if the impending phase of relapse, overdoses, and suffering really was necessary and repeatedly expressed a wish “that someone had intervened” (Harper:26).

For others, it seemed that the positive associations to substance use accumulated in the early phases of addiction needed to be outweighed by the negative associations gained after the drugs changed from cure to cause before quitting was an option:

*When I was younger*,* I had more positive experiences. And since I’ve grown older*,* I’ve gotten more negative experiences*,* so I’ve also come to associate it with displeasure (Kimberly:15).*

For Kimberly, the turning point where the negative experiences outweighed the positive came abruptly when her stepfather, whom she considered the personification of her substance use, sexually abused her: “and then I got fed up with him and the whole situation” (Kimberly:25). She managed to remain drug-free from that point onwards.

From Marlon’s account, it seemed that time had degraded the sustainability of “it” and, in its collapse, he saw an opportunity to leave:

*It is not like it used to be. But it’s a good thing for me*,* so I can quit*,* you know [chuckles]. I was out a while back*,* recently*,* in August/September. It was hell. […] You can’t live as a criminal anymore. Plus*,* these wee ones in charge of their territories*,* threatening and carrying on*,* you don’t get a stake in the market*,* you’re older now*,* the police are on you all the time. I’m done with it. I’ll head out and get a job somewhere. It’s better (Marlon:45).*

For these participants, their MD had provided a vulnerability for their substance use to utilise to embed itself in all of life’s domains. Quitting the substance use without also tearing down all domains which it had come to support was impossible. Only when this self-sustaining ecosystem fell into ruins, was it possible to build something new.

### Treated but not cared for

In this theme, patients’ experiences with being treated for COD are illustrated. Most patients were aware that their inability to leave “it” until their lives fell apart likely meant one of three outcomes: death, prison, or the FMHS. In contrast to the alternatives, receiving treatment in the FMHS was often considered a salvation. Still, a recurring sentiment in participants’ experiences of treatment was an unmet desire to be genuinely cared for, and instead feeling “simply” treated by an inflexible institution.

All had, in some way, experienced a disconnect between their treatment needs while being stuck in the middle of “it”, and the help they had been offered. In reflecting on it, they now wished that the interventions they had received had been more tailored to their specific needs. Paradoxically, this meant more coercive measures to some, and a gentler understanding to others. Lenny’s transcript contained frequent examples of his frustration with receiving standardised, impersonal treatment that missed the mark: “It isn’t care at all, just treatment. Like classes and stuff” (Lenny:17). Lenny made a clear distinction between being cared for and being treated. SUD and COD interventions, both past and present, had been too rigid to meet the unique combination of issues facing Lenny specifically and “would only be about diagnosis and so on” (Lenny:24).

Like Lenny, Kimberly found her diagnoses reductive. For her to benefit from treatment, she needed to talk in broader terms about her mental health than what could be confined within the rigid diagnosis parameters.

*I’ve gotten my diagnosis paranoid schizophrenia. I agree with schizophrenia*,* but not paranoid. But that’s the diagnosis I’ve gotten. Then I’ve also gotten atypical autism or autism diagnosis. But I don’t recognise myself in that one (Kimberly*: *11:19).*

By partially rejecting the diagnosis labels, Kimberly showed how poorly they defined her. Like the other participants, there were aspects in her diagnoses Kimberly recognised and others she rejected. Having their mental health reduced to and forcedly fitted into a diagnosis label left patients feeling unseen and de-personified.

Estrangement with diagnosis labels created a disconnect between patients and their disorder and, consequently, with the purpose of their stay in the FMHS. Rather than working on “mental vulnerabilities” that they themselves recognised, they often effectively dismissed the FMHS label. Charlie considered his diagnosis not as an indication of a persistent disorder he needed to manage, but as something transient – a label the FMHS had given him but that he did not internalise: “It’s gone now away. I’m fine now. Just waiting to be transferred out of here” (Charlie:17). Charlie saying he was “just waiting” indicated that additional social and psychological interventions were wasted on him. There was nothing left for FMHS to treat. His insistence that he was “fine now” and that his diagnosis was “gone” reflected a common urge to say whatever was necessary to expedite discharge from an indefinite treatment period.

Fear of the review board extending their treatment period also demotivated patients from engaging in treatment in meaningful ways. Benjamin felt bewilderment over the length of stay for some and was ready to do and say anything to avoid it himself:

*I know people [in FMHS]*,* they’ve been here for 15*,* 17*,* 12 years and such. When I hear these numbers*,* I get scared you know? I think ‘that can happen to me too’. It’s just because they’re idiots. Each time they get the chance*,* they start using again. That’s why they’ve been stuck for so many years. I am not that stupid. […] I am never coming back here. Not a chance. You get me? This is like a lifetime sentencing with a chance of being pardoned. It’s what it feels like (Benjamin:14–16).*

A shared experience between participants was that the impersonal and rigid treatment design in FMHS required them to conform to an often ill-fitting definition of their MD. Across all transcripts was the ubiquitous awareness that if you refused the institution’s definition of your disorder(s) or if you shared aspects of your mental health or your addiction that the institution was unaware of, the discharge review board would learn of it and your length of stay would be extended.

*Yeah*,* most patients lie about that. Everyone lies about that. Ain’t no one wants to expose themselves to the [FMHS]*,* I feel. It’s hard*,* man. People don’t dare to talk here. It’s not like in the 12-step program at the treatment centres. [Clinic staff here] is all like ‘wow*,* that much? Oh fuck!’ People get scared to talk*,* you know*,* about their disorder*,* about their addiction*,* about their criminal past. Just to avoid consequences*,* you know. […] Because it all gets back to the discharge review board (Marlon:5).*

Here, and elsewhere in Marlon’s transcript, there was a sense of exasperated resignation. Since he had personally surrendered the edited façade that other patients maintained in the FMHS, he was mentally preparing for a life of relapse and recommitment – a life he believed had driven others to suicide: “so he hanged himself. […] Its hard being recommitted. It is the hardest thing” (Marlon:10).

For Marlon and most other participants, the FMHS system possessed an often impossibly inflexible authority over what COD treatment should consist of, how it should be administered, and what defined successful treatment outcomes. Rigid treatment design left several participants feeling treated but not cared for, and rigid definitions of successful treatment outcomes consigned patients to a terrifying Sisyphean cycle of relapse and recommitment.

### Comprehending fragments of the self

Positioned within a discourse on free will and determinism were the participants’ efforts of sense-making of amoral behaviour perpetrated by a moral self. They resolved the discrepancy between who they were and who they had been by conceptualising two selves: user and victim.

The participants’ sense-making process was framed within an existential query which was best exemplified by Marlon when he discussed his inability to avoid relapse:

*You can’t control the human being. You can’t control anyone. Unfortunately. We’re all human beings*,* you know*,* we’re not robots (Marlon:39).*

Marlon also exemplified how participants’ sense-making of their COD often positioned them along the biological determinism – behavioural voluntarism dichotomy: “if you’ve lived a life like I have, then you’re born with a diagnosis” (Marlon:31). Similarly, Jean understood her alcohol addiction as an inevitability attributable to her genes or elements in her childhood:

*Because mom was an alcoholic. So I had it at home. […] Alcoholism was part of my home*,* so to say*,* and so you felt that ‘if she drinks*,* I might as well start’ (Jean:1–4)*.

Jean provided several examples of exercising her free will in choosing to not do hard drugs or in avoiding criminal elements, but simultaneously understood her alcohol abuse as inevitable due to her familial exposure to it. Her eclectic perception of agency illustrated the flexibility shown by many participants in making sense of their amoral past while maintaining a grip on themselves as moral selves.

Recurringly, participants’ way of phrasing indicated how they positioned themselves as subjected to, as opposed to active agents in, the events that unfolded around them: “it was the Spice that brought me here…” (Harper:12); “you’d drink too much and then you’d end up in a fight” (Ahmed:05); “[residential treatment] was a good place to end up at” (Jean:34.5); “yeah, it turned out that way” (Jean:9.5).

Marlon and Lenny also framed themselves as agentless subjects to life’s throes. They both had been raised in similar, religiously conservative environments, and had felt unable to control themselves when introduced to Sweden’s liberal culture: “I was young and had everything I wanted. But I was uncontrollable” (Marlon:22); “I had big addiction. Nothing could stop me that time.” (Lenny:4). Repeated descriptions of being “uncontrollable” and that “nothing could stop” them revealed a significant aspect of how they understood their past behaviour – the onus of control rested with someone else.

Concerning the origins of their addiction, participants’ victim cognition crystalised. There were tendencies to find a way to assign responsibility elsewhere and cast themselves in the role of victim. Harper’s, Ezekiel’s and Finnegan’s transcripts provided windows into this:

*Also*,* I was actually tricked the first time. I wanted a cigarette*,* and I got cannabis. At first*,* I didn’t know*,* but then it tasted different. Then the guy said that ‘it’s only cannabis*,* it’s fine.’ But I noticed it calmed me down. But I thought it was pretty shitty. I was tricked into it. Then I kept going on my own*,* that wasn’t his fault (Harper:02).*

Harper recognised that it “wasn’t his fault” but still highlighted the notion that she was a victim of trickery and that her addiction, and all problems caused by it, stemmed from this one moment. Ezekiel explicitly stated that his substance use “wasn’t exactly my fault” (Ezekiel:05), and the process “was automatic” (Ezekiel:05) implying that he played no active part in it.

The sense of confusion that was often present in the participants’ attempts to comprehend an amoral past marred with behaviour that seemed foreign to their current, moral selves indicated a sense of fragmentation of the self. Trying to make these fragments fit together, into a sense of self that could incorporate the SUD, the criminality and their current and capable selves often emerged as the conception of two distinct identities: one responsible for the substance use and the ensuing violence, and another without agency who was victimised by the growing addiction. Resolving this identity confusion by conceiving two selves was strikingly in Finnegan’s transcript:

*I was two people. One was an addict*,* and one took care of himself*,* spent time with his family*,* and helped out and such. So I lived a double life (Finnegan:5).*

By contrasting the two selves, Finnegan created a framework to comprehend his fragmented experiences. He ascribed to the “addict self” qualities antithetical to his moral identity – not caring for himself or his family. As his substance use intensified, so did his sense of fragmentation of the self and his “addict self” gradually came to outgrow his prosocial aspects. By attributing destructive behaviour to this “addict self,” he was able to make sense of actions that felt disconnected from his core sense of self while simultaneously maintaining his moral identity.

Marlon’s transcript suggested that he too struggled to reconcile his moral self with his amoral, violent past and had come to question his degree of agency. Like other participants, his experience of a fragmentated self manifested in his attribution of SUD and violence to a separate identity. Marlon believed that addiction was innate and that, when externally unrestrained, a violent version of himself emerged. In sober moments, he had wrestled for control with this other self and eventually managed to lull it back to sleep with cannabis. He still referred to himself as “a dangerous old man” (Marlon:14) implying that he had not changed, just aged. His transcript contained several instances of how this version of himself still existed within him:

*No*,* I just got [mild medication]. Not [strong medication] or anything like that. I was good on [strong medication]*,* but they didn’t want to wake me. Better I stayed slumbering (Marlon:23).*

Marlon regarded his dangerous self, the self to which he attributed the drug use, the criminality, the violence, and the suffering, not as gone but as dormant. Treating him with narcotic drugs risked awakening this other self. Strikingly, Lenny conceptualised his sense of a fragmented self very similarly – as one self being awake while the other was dormant: “but after I woke up, after the clinic made me wake up: ‘what have I done? Why I drink?’” (Lenny:6).

Together, participants’ lack of control, their perception of themselves as victims, the conceptualisation of a second self to resolve the dissonance between their role as victim and their amoral actions, along with the sensation of “waking up” and having the other self fall dormant, placed their time in the FMHS in a surreal light. Gael feared that, after his time at the clinic, his other self – the addicted self – would be there waiting for him.

*It feels like… It feels like an entirely different person used drugs*,* compared to who I am now. […] It’s easy to say that when you’re in this environment. This is artificial. It’s much more difficult to manage when you’re out (Gael:17).*

Participants’ reports frequently showed a sense of estrangement to their past behaviour and a recurring notion of lacking agency in the development of their COD revealing a sense of fragmented self. Struggling to make sense of this selective disconnect with themselves led to the conception of two selves, allowing many to compartmentalise their amoral actions and still maintain a moral identity.

## Discussion

The aim of this study was to describe and interpret the experiences with COD among patients in Swedish FMHS, with particular focus on disorder development and treatment. To our knowledge, this is the first IPA of patients’ own sense-making of their experiences with COD in this context.

The participants made sense of their substance use as an effective and desperate solution to an acute problem. While aware of the spiral of worsening mental health, criminality and violence that the substance use had led to, many still defended their choice, emphasising the need for an immediate solution. The transcripts often contained a sense of estrangement from the inner processes that expressed themselves as self-harm or aggression. Some had felt vague and overwhelming sensations, sometimes described as a “buzzing” in their minds, which would later be diagnosed as conditions such as borderline personality disorder. The “missing tool” that many lacked was likely an expression of alexithymia – a multifaceted trait defined as having difficulty identifying and describing feelings [44]. Alexithymic traits typically develop during critical developmental periods in childhood, when attachment relationships form to provide the foundation for emotional competence [45]. Secure attachment enables children to recognise, label, and regulate emotional states through caregiver interactions [ibid]. However, early-life trauma – highly prevalent in FMHS populations [4] – disrupts this process and causes children to disconnect from emotions as a survival strategy [46]. This developmental disruption can sometimes manifest as alexithymia in adolescence and adulthood. These findings are supported by past studies demonstrating that people with alexithymia drink more than healthy controls, likely as a means to cope with negative affect [47], and that the deficits in social functioning resulting from alexithymic traits are more pronounced in people with SUD [48]. Furthermore, deficits in emotion recognition and regulation may contribute to the aggressive and violent behaviour characteristic of FMHS populations [49, 50]. For adolescents with early-life trauma and alexithymic traits who lack the neurobiological maturation necessary to manage traumatic experiences or early-onset MDs, substances offer a readily accessible means of managing intense, unnameable dysphoria (the “buzzing”). It is not surprising then, that many found substances to be such an efficient and legitimate tool.

As participants’ addiction deepened, their distinction between SUD, MD, and lifestyle blurred. The participants did not consider their MD and their SUD as two co-occurring disorders, but mutually reinforcing expressions of an underlying pathology, poorly contained within neat diagnostic boundaries. Instead, they referred to “it”, indicating a kind of substance-suffused ecosystem that both sustained them while simultaneously compelled them to sustain it, leading to the abandonment of pro-social attachments and eventually to violent crime. In all likelihood, the participants’ emotional and/or cognitive vulnerabilities due to past trauma or undiagnosed MD served as a catalyst for the psychological and chemical addictive effects of substance use, facilitating the expansion of SUD’s network of roots. To those embedded in “it”, treatment interventions that asked for cessation of substance use actually asked them to tear down “it” all: their social life, romantic life, source of income, pain and mental health management, and their identity. For these participants, this was asking too much. Only when the integrity of this ecosystem had degraded and collapsed was it possible to rebuild.

Participants’ notion of “it” as an ecosystem resonated with “out there” – an ecology of addiction described by Weinberg [51]. “Out there” was conceived of as a degraded and dangerous wilderness in contrast to remaining in treatment. Treatment clients used this juxtaposition to facilitate the reconciliation of two opposing truths: being compelled by their addiction and being able to manage addiction by effortful control. Amoral actions taken “out there” were condemned as the requirements of a hostile ecology and allowed the client to claim the level of agency needed to engage in therapy, while simultaneously perceiving themselves as victims of a compulsive disease [18, 51]. Participants in this study showed a similar tendency to conceive of their “addict self” – responsible for amoral actions – as separate from their current selves – amenable to treatment. Solving this conceptual puzzle was facilitated by the notion of “it” as a separate world inhabited by the “addict self.” This separation allowed participants to attribute their amoral past to the “addict self” within “it” while preserving their current, moral self as capable of change.

Participant’ experiences with treatment varied. Participants’ challenges with accessing siloed treatment in the past join a chorus of critique against outdated treatment provision [52–56] and currently, most felt that conventional treatment provision left too little room for their comprehension of their treatment needs. Rigid treatment approaches that emphasised diagnosis labels – sometimes seen as inaccurate by patients – limited engagement in treatment, as has been evidenced in previous studies [57, 58]. In FMHS, this rigidity, coupled with discharge being contingent on treatment progression, led to censorship and alienation from treatment. The resulting “as if” treatment, based on edited presentations, often stalled treatment progression. These results underline the need for forensic treatment to align treatment practices with patients’ definitions of their disorders and treatment needs [10, 11]. If treatment institutions’ design intimidates patients into censorship, as suggested by this study and others [31], it risks obscuring treatment needs and undermining the effectiveness of interventions.

The participants’ experiences of feeling “treated but not cared for” and their need to maintain façades to expedite discharge point to fundamental problems with how services are structured. To address the censorship that the power imbalance between patient and institution induces and the alienating effect that diagnosis focused treatment has, the FMHS would benefit from implementing trauma-informed treatment approaches [21]. Trauma-informed care provides a framework for recognising that trauma underlies much of what presents as pathology in the FMHS [59]. The five principles within this framework – safety, trustworthiness, choice, collaboration, and empowerment – would directly address the power dynamics experienced by many participants. If patients had a greater degree of agency in their treatment planning and felt free to share their actual experiences of “it” without fear of extended sentences, the therapeutic relationship could shift from performative compliance to authentic engagement. Previous studies show marked benefits in treatment outcomes – fewer violent incidences, lessened engagement in substance use, improvements in psychiatric symptoms – after implementing trauma-informed care [20]. For the participants in this study, who understood their substance use as interwoven with trauma and mental health in the complex ecology of “it,” treatment that acknowledges this interconnection rather than targeting isolated diagnoses could be transformative. Identity transition – that crucial shift from “addict self” to something new – cannot happen through force or fear. It requires the treatment community to collaboratively construct new narratives with patients, narratives that make sense of past trauma, acknowledge ongoing vulnerabilities, and imagine different futures [60].

### Limitations

Certain limitations should be noted. Firstly, the IPA does not necessarily aim to generalise findings beyond the analysed sample. There is no guarantee that the experiences presented here are found elsewhere, but that does not make them any less real. The study’s purpose was to interpret the lived experiences shared between participants while giving voice to their unique stories told within the context of Swedish FMHS. While the sample size exceeds typical IPA recommendations [39], the brevity of some interview – potentially due to substance related memory deficits, or due to the participants’ experience with clinical assessments – allowed for this larger sample without compromising analytical rigour [61]. Participating patients likely had better cognitive functioning and ability to mentalise about their experiences than those who declined or were ineligible for participation – a selection bias that must be considered. Another possible bias is that participants may have exaggerated or understated elements of their experiences due to their desires to elicit sympathy, scepticism toward the research project, or impaired meta-cognition [51]. Such biases are particularly relevant in forensic contexts where impression management is prevalent [62]. However, the phenomenological approach means studies the phenomenon as it is experienced by the participant [39], making these possible motives part of the participants’ experiences with COD. A further consideration concerns the directionality of participants’ narratives and the institutional context in which they were constructed. Given the powerful, coercive nature of the FMHS where discharge is contingent on treatment progression and compliance, it is likely some participants had adopted a “treatment-sanctioned” vernacular when making sense of their COD and communicating their self-perception. This makes it difficult to confidently separate participants’ authentic perspectives from institutionally shaped discourse. However, this limitation adds nuance and weight to the “treated but not cared for” theme, as it demonstrates the pervasive influence that the institution has over patients’ narratives. Finally, consistent with hermeneutic phenomenology, the results presented here are the authors’ interpretations of the patients’ lived experiences – other interpretations are possible.

## Conclusions

Based on this IPA of 13 participants’ lived experiences with COD in Swedish forensic psychiatry, several potential implications for clinical practice emerge:

- **Comprehensive treatment of “it”**: Treatment would benefit from moving beyond diagnostic models toward a comprehensive approach that addresses all life domains that constitute “it”, as setbacks in any domain formerly part of “it” risk inducing relapse and reanimating the ecosystem. Person-centred treatment design that aligns with patients’ understanding of their psychological vulnerabilities will likely limit the risk of censorship and make treatment feel more authentic and less “as if”.
- **The trauma-informed care perspective**: Considering the prevalence of early-life trauma among FMHS patients and the current study’s findings on how substance use served as a legitimate tool in lieu of healthy alternatives for trauma-management, adapting a more trauma-informed treatment approach could be beneficial. Without compromising security concerns, the FMHS could consider adopting the key principles of trauma-informed care: minimizing re-traumatization, recognizing trauma’s impact on emotional regulation, and creating spaces for reflection.
- **Sustained recovery**: Participants conceptualised the “addict” fragment of the self as dormant rather than gone and some feared it awaited them upon discharge. Comprehensive discharge planning with robust support during the transition into outpatient care is critical for helping patients navigate a more independent life without reverting to old routines and reanimating “it”.

These findings suggest that effective COD treatment in the FMHS must be flexible and responsive to patients’ lived experiences. FMHS and outpatient services are recommended to move beyond rigid diagnostic approaches toward trauma-informed care that addresses the complexity of “it” and supports sustainable recovery through comprehensive discharge planning.

## Data Availability

Due to the sensitive nature of the data and the risk of identifying participants, data will not be made available. Contact the corresponding author for more information.

## Abbreviations

COD: Co-occurring disorders
SUD: Substance use disorder
MD: Mental disorder
FMHS: Forensic mental health services
IPA: Interpretative phenomenological analysis
